# A Case of Uterine Cancer

**Published:** 1880-10

**Authors:** Charles T. Parkes

**Affiliations:** Prof. Anatomy Rush Medical College and Physician in Charge, and Obstetrician to St. Joseph’s Hospital, Chicago


### ARTICLE IV.

A CASE OF UTERINE CANCER. By CHARLES T. PARKES, M.D.

Prof. Anatomy Rush Medical College and Physician in charge, and Obstetrician to St. Joseph's Hospital, Chicago.

The fact that Listerism robs abdominal surgery of all its former terrors and fatality, frees the surgeon's mind from great anxiety with reference to such operations and enables him to give favorable prognoses in the most formidable and unfavorable cases applying for aid, is well established by the results of the practice of such men as Lister, Wells, Keith, Thornton, Volkman, and others.

Antiseptic exploratory section of the abdominal walls, can safely and properly be made use of, in all such cases where external manipulation and examination fail to determine absolutely the condition of the parts affected with disease.

Unrelieved intestinal obstruction and perforating wounds of the abdomen heretofore considered necessarily fatal, will in time be treated by such section for relief with the certainty of complete success following the interference, if done under full antiseptic precautions.

The following interesting case is one of the many isolated examples, all the time coming to the notice of the profession, in proof of the safety and innocuousness conferred by the method of Lister upon abdominal operations, and the successful results of which urge upon the profession the necessity of becoming more thoroughly acquainted with, and more carefully practicing antiseptic procedures.

This history of the case is transcribed from the records as taken by Dr. E. M. Landis, house physician to St. Joseph's Hospital, and confirmed by myself.

Mrs. Anna Eckhart, age 38, born in Germany, married 15 years, and, including three miscarriages, confined eleven times in eleven years—eight living children. No hereditary disease or predisposition thereto in her family that she is cognizant of. Father died at 75 years of heart disease, and mother still living at also 75 years. Grandparents also lived to old age. Up to the time of her present illness, was always healthy, and not unduly exposed by hardship or other conditions, to circumstances favorable to contraction of disease. Weighed 197 lbs. when in health.

The patient was admitted to St. Joseph's, July 11th 1880, suffering from hœmorrhages from the womb, with severe pain in the region of the pelvis, a constant discharge of a large amount of foul smelling ichorous pus from the vagina, and other history of malignant disease of the womb.

Digital examination disclosed the upper half of vagina filled with a soft mass of foreign growth as large as an orange, very friable, and easily made to bleed profusely. The finger could be made to pass beyond the growth, along the anterior vaginal wall, into a cavity, the top of which could not be reached. Posteriorly the touch was stopped at the cervico-vaginal junction. The speculum merely confirmed the touch, showing the growth to be cell proliferation from a cavity above into the vagina.

*Diagnosis.*—Cancer of the uterus, probably growing from the posterior wall of body and cervix, probably epitheliomatous.

*Treatment.*—Sol. ferri persulphate applied to control bleeding, and repeated irrigation with 1 to 40 solution of carbolic acid to control fetor; tonics and iron to correct anæmia and increase strength. Patient shows well marked cachexia, wax-like ears, quasi-œdematous appearance of the skin.

*Previous History of Disease and Treatment.*—Up to July 20th, 1879, she had been apparently in good health. Her menses had been regular and orderly until fourteen days previous to the above date. On July 20th, 1879, she had a severe hæmorrhage from the womb, and at first thought she was having a miscarriage. Fourteen days later, or at the time of her menstrual flow, she had another severe hæmorrhage. From this time bleeding was constant in a greater or less degree, never very profuse, and unaccompanied with pain. Sunday, four weeks previous to Christmas, 1879, she consulted Dr. Geiger, of Chicago, who, after examining her, referred her at once to Dr. A. R. Jackson, of Chicago. Dr. Jackson informed her that an operation was required for her relief.

She was then admitted to the Mercy Hospital, of Chicago, on January 3d, 1880, and on January 8th, at three o'clock p. m., Prof. E. W. Jenks performed some operation about the womb, before the class of the Chicago Medical College.

She says she was told that the Professor removed her womb so completely as to leave remaining only the broad ligaments, ovaries, and a thin portion of the top of the womb, or, as she expresses it, "bis auf die Mütter Trumpeten."

After recovering from the effects of the operation, she returned to her usual duties.

Her menstruation came on eight days after the operation, and seemed normal and regular, until the first week in April, 1880, or about three months after the operation, when a slight hæmorrhage and a foul discharge appeared, which, since then, has been constant to the date of present examination, July, 1880. In May, 1880, she first began to suffer pain, which gradually became unbearable without full doses of morphine.

Three weeks previous to admission into St. Joseph's Hospital, she had a third and the most profuse hæmorrhage she has yet suffered. Began to lose flesh and strength from the very commencement of her trouble—still she is far from being emaciated.

The patient has consulted Prof. Jenks several times at his office since the performance of the operation.

The patient was very anxious to undergo any operation that promised any relief whatever from her trouble, and expressed herself as feeling convinced that she could not expect any probability of permanent relief unless the womb and its appendages were removed through the abdominal walls. She was convinced that no operation through the vagina could cure her. The patient was thoroughly acquainted with the fact that no operation promised her radical cure; that it was extremely doubtful whether total extirpation could be at all safely performed after abdominal section, owing to the extensive involvement of the pelvic organs in the disease, but that such section could be safely done, and would settle the possibility of relief from a radical removal; and further, some operation, either total extirpation, if warrantable, by means of abdominal section, or curetting away the mass per vaginam, was necessary to give her a period of relief at least from her pain, and septic inoculation from the foul discharge.

She expressed herself prepared to submit to whatever we concluded to do, and after due consideration, on July 22d, 1880, the patient was etherized, and the following procedures carried out under full and complete antiseptic precautions, in the presence of Drs. Gunn, Bogue, Fitch, Fenger, Etheridge, Bartlett, Landis, and others.

An incision four inches in length was carried through the different layers of the abdominal walls down upon the peritoneum.

This being opened, and the intestines pushed out of sight, the following condition of the pelvic contents was found :

Practically, the cavity of the pelvis was found obliterated and filled with a dome-like development of foreign growth, which occupied all the irregularities of that cavity, and reached to an elevation on a line with the inlet of the pelvis. Posteriorly there was no cul-de-sac of Douglas. Centrally the entire body of the uterus and its appendages were intact, and raised aloft upon the undergrowing mass of cancerous infiltration. Laterally the mass was as high as the pelvic brim. Anteriorly the top of the bladder only was visible. Several enlarged glands of considerable size were found laterally and posteriorly.

An attempt to remove any such mass of disease was clearly unwarrantable.

The abdominal incision was closed by interrupted sutures and gauze dressings applied with retentive bandage. The patient was then placed in Sim's position, the vagina well opened and retracted, and the foreign mass as completely removed as possible with the scissors, curette, finger and Pacquelin cautery. All friable tissue within reach was carefully and radically scraped away. Thanks to the cautery, this was accomplished with extremely little hæmorrhage.

This operation cleared the vagina entirely, leaving a very shallow rim of what seemed to be a remnant of the cervix uteri at its upper termination. Above the vagina was left an immense cavity, the anterior wall of which seemed to be formed by the posterior wall of the bladder, as the sound in the bladder could be easily located by the finger in the cavity ; the roof was formed by the parts already seen and felt through the abdominal walls ; posteriorly the finger in the rectum could be located through the cavity. The patient had not a single unfavorable symptom after these surgical procedures, either of which is entitled to an appellation of more gravity than usually goes with the word "simple." On the eleventh day she was out of bed, and on the twelfth she walked down stairs. The abdominal wound healed firmly without any local trouble except a little irritation around one of the lower sutures. The patient improved in strength and appetite, and had no pain and no foul smelling discharge. This improvement was only transient, however, for during the month the discharges again became offensive, and the pain returned, so that now, September, 1880, although suffering less than previous to the operation, and although less offensive to herself and others, the signs are none the less positive that the disease is steadily progressing and will be fatal in its issue.

The parts exposed by the abdominal section were examined by several of the gentlemen present, and all remarked the natural and healthy aspect of the body of the uterus, including certainly a small portion of the cervix, as the broad ligaments were intact. Evidently the body of the uterus had never been encroached upon nor invaded by the disease, which had seemingly developed in and about the cervix, and in its development pushed everything else before it.

Since performing the above operation, much to my surprise, I find that the operation performed upon this patient in Mercy Hospital on January the 8th, 1880, has been classified, and referred to in a number of medical periodicals, as one of *recovery* from total extirpation of the uterus per vaginam. Such reference to this successful and radical operation may be found in the *Chicago Medical Gazette*, No. 3, February 5th, 1880, page 55; also in the report of Mercy Hospital Clinics, March 5th, 1880, No. 5, page 116 of same journal; also the last number of Braithwaite's Retrospect Gynæcological Reviews.

Any statistics based upon such report will, of course, possess more than the usual unreliability belonging to an array of figures used to back up any positive course of treatment, or from which conclusions may be drawn.

---

THE valuable papers abstracted in this JOURNAL from proceedings of the American Medical Association, on "Rest after Delivery" and "Treatment of Peritonitis," were from the pen of Dr. Tauksyn.

